# Recalculating the Net Use Gap: A Multi-Country Comparison of ITN Use versus ITN Access

**DOI:** 10.1371/journal.pone.0097496

**Published:** 2014-05-21

**Authors:** Hannah Koenker, Albert Kilian

**Affiliations:** 1 Johns Hopkins University School of Public Health Center for Communication Programs, Baltimore, Maryland, United States of America; 2 Tropical Health LLP, Montagut, Spain; Queensland University of Technology, Australia

## Abstract

Use of insecticide treated nets is widely recognized as one of the main interventions to prevent malaria and high use rates are a central goal of malaria programs. The gap between household ownership of at least one ITN and population use of ITN has in the past been seen as evidence for failure to achieve appropriate net use. However, past studies compared net use with ownership of at least one net, not access to sufficient nets within households. This study recalculates the net use gap in recent large household surveys using the comparison indicator of ‘access to nets within the household’ as now recommended by Roll Back Malaria and the World Health Organization. Data from 41 Demographic Health Surveys (DHS) and Malaria Indicator Surveys (MIS) (2005–2012) in sub-Saharan Africa were used. For each dataset three indicators were calculated: population access to ITN, population use of ITN, and household ownership of at least one ITN. The ITN use gap was expressed as the difference between one and the ratio of use to access. The median proportion of users compared to those with access was high, at 82.1%. Even at population access levels below 50%, a median 80.6% used an ITN given they had access, and this rate increased to 91.2% for access rates above 50%. Linear regression of use against access showed that 89.0% of household members with access to nets used them the night before. These results clearly show that previous interpretations of the net use gap as a failure of behavioral change communication interventions were not justified and that the gap was instead primarily driven by lack of intra-household access. They also demonstrate the usefulness of the newly recommended ITN indicators; access to an ITN within the household provides a much more accurate comparison of ITN use than ownership.

## Introduction

Use of insecticide treated nets (ITN) is widely recognized as one of the main interventions to prevent malaria and high use rates are a central goal of malaria programs. The two main indicators to assess outcomes have been household ownership of at least one ITN and population use of ITN the previous night. Consistently, evaluations have found a significant gap between these indicators with ITN use always much lower than ownership of at least one ITN [1]–[9] and this has been interpreted as evidence of failure to achieve appropriate net use or as a failure of behavior change communication (BCC) to adequately improve ITN use rates [2], [10]–[12]. Even very recent publications have continued in this trend [13], [14] calling for more educational campaigns to close the entire assumed gap. However, the comparison of ownership to use is misleading and inaccurate for two reasons: first, the denominators of the two indicators are different; second, the ownership indicator does not account for insufficient intra-household net saturation, i.e. some household members will not able to use an ITN simply because it is not there, irrespective of motivation to use.

ITN use is affected by many factors, including seasonal perception of risk, mosquito biting density, perceived comfort, household composition, physical space constrictions [15]–[21], and in some cases where a variety of nets are available, net preferences [22]–[24]. However, several authors have pointed out that the main reason for non-use is lack of access to a net [25] and having enough nets for all within a household is the strongest determinant of net use [26], [27]. Accordingly, adjustments were made for ownership of nets within the household by restricting analysis to net-owning households [26], [28] or including the variable of at least one ITN for every two household members [29]. Vanden Eng [30] introduced a framework of four categories assessing whether individuals were living in households where a) nets were not owned, b) nets were owned but not hung, c) nets were hung but not used, or d) nets were used, in an effort to specify whether non-use of nets was behavior- or access-driven. However, this framework still did not account for whether there were enough nets in the household. Thwing [31] and West [32] reported on the percentage of households with enough nets to cover all sleeping spaces, but did not conduct use analysis for these households. The most recent suggestion is presented by Singh et al. [33] in a review of ITN use during pregnancy which introduces the indicator “use of an available net” which here refers to ITN use by a pregnant woman if the household owns at least one ITN. However, none of these approaches is able to clearly define the behavioral part of the gap between ownership and use as they fail to clearly define access to a net or ITN within the household.

Acknowledging the shortcomings of the existing two indicators for ITN programs, the RBM Monitoring and Evaluation Reference Group (MERG) reviewed them in 2010 and in 2011. The group recommended the addition of two new indicators [34], namely the proportion of households with one ITN for every two people (“household access”) and the proportion of the population with access to an ITN within the household (here referred to as “population access” or simply “access”) with the assumption that an ITN protects on average two people. These new indicators allow for direct comparison against household ownership and population use, respectively, aligning with best practice for using appropriate comparators for assessing health program implementation [35]. Kilian and colleagues [36] recently described in detail how these indicators can be applied for a comprehensive ITN program analysis using Nigeria as an example. Recent WHO World Malaria Reports also presented a generalized analysis of population ITN use compared to population ITN access [37]–[40]. The aim of the present study was to recalculate the net use gap – the relationship between access and use rather than ownership and use – using data sets from the last seven years and the updated comparison indicator of ‘access to nets within the household’ as recommended by RBM and WHO.

## Methods

Data from 41 DHS and MIS surveys (2005–2012) in sub Saharan Africa were used which were downloaded with permission from the Measure DHS web site. For each dataset three indicators were calculated: individual access to ITN within the household, individual use of ITN the previous night, and household ownership of at least one ITN. The ratio of population ITN use to population ITN access within the household was calculated and is referred to here as the use:access ratio. The ITN use gap is therefore calculated as 1 minus the use:access ratio. The ITN variables were used rather than LLIN due to the fact that in the earlier surveys some conventionally treated nets were still present. The majority of ITNs in this analysis, however, are LLIN.

Data management and analysis was done using STATA version 12 (STATA Corporation, College Station, Texas, USA) or Excel 2010 (Microsoft Corporation, Seattle, Washington, USA). All analyses accounted for survey design including sampling weights where applicable using the survey command family in STATA.

The survey indicator of access to ITN within the household was calculated from the datasets of individual household members as recommended by MERG [34]. First, an intermediate variable of “potential ITN users” was created by multiplying the number of ITN in each household by a factor of 2.0. In order to adjust for households with more than one net for every two people, the potential ITN users were set equal to the de-facto population in that household if the potential users exceeded the number of people in the household. Second, the population access indicator was calculated by dividing the potential ITN users by the number of de-facto members for each household and determining the overall sample mean of that fraction.

Use of an ITN the previous night was calculated for each *de facto* member of the household, i.e. those present in the house the previous night, as recommended by MERG using the listings of net users from the net roster [34]. Household ownership of at least one ITN was also calculated for each dataset based on the number of ITN observed in the household and defining an ITN as a long-lasting insecticidal net (LLIN) identified by its label or a net that was treated with an insecticide within the last 12 months.

Linear regression was used to describe the relationship between use, ownership and access and in order to acknowledge the fact that no use is possible without access or ownership, all models were run with a “no constant” constraint.

## Results

Details of the 41 datasets are provided in Table 1. Surveys were conducted between 2005 and 2012, and consisted of 28 DHS (57%), twelve MIS (41%), and one Anemia and Parasitemia Survey. A total of 28 countries in sub-Saharan Africa were represented, with sixteen surveys (39%) from West Africa, fourteen (34%) from East Africa, five (12%) from Central Africa and six (15%) from Southern Africa.

**Table 1:** Access, use, and ownership of ITNs by survey.

Country | Survey | Year	% of households owning at least 1 ITN	% of population with access to an ITN within their own household	% of population that used an ITN the previous night	Ratio of use to access
Angola MIS 2006–2007	27.5%	14.5%	11.9%	0.82
Angola MIS 2011	34.5%	19.0%	18.9%	0.99
Benin DHS 2006	24.5%	14.7%	14.7%	1.00
Burkina Faso DHS 2010	56.9%	36.1%	31.5%	0.87
Burundi DHS 2010	52.0%	39.1%	37.8%	0.97
Burundi MIS 2012	66.0%	46.0%	48.6%	1.06
Cameroon DHS 2011	36.4%	10.8%	7.6%	0.71
Cote d'Ivoire DHS 2012	71.7%	49.0%	33.2%	0.68
DRC DHS 2007	9.2%	4.2%	4.3%	1.03
Gabon DHS 2012	44.1%	26.9%	26.7%	0.99
Ghana DHS 2008	41.7%	30.1%	20.9%	0.69
Guinea DHS 2005	3.5%	1.5%	1.1%	0.77
Kenya DHS 2008	55.7%	42.3%	35.1%	0.83
Liberia MIS 2009	47.2%	25.4%	22.8%	0.90
Liberia MIS 2011	49.7%	30.8%	32.1%	1.04
Madagascar DHS 2008	57.0%	34.7%	36.6%	1.05
Madagascar MIS 2011	80.5%	57.3%	68.4%	1.19
Malawi DHS 2010	56.8%	37.6%	29.0%	0.77
Malawi MIS 2012	55.0%	37.2%	40.9%	1.10
Mali Anemia & Parasitemia 2010	85.9%	61.6%	56.2%	0.91
Mali DHS 2006	50.0%	29.7%	21.4%	0.72
Mozambique DHS 2011	54.7%	37.0%	29.4%	0.80
Namibia DHS 2006	20.2%	12.8%	5.5%	0.43
Niger DHS 2006	43.0%	19.6%	4.4%	0.22
Nigeria DHS 2008	8.0%	4.8%	3.2%	0.68
Nigeria MIS 2010	41.5%	28.7%	23.3%	0.81
Rwanda DHS 2007–2008	55.6%	38.1%	39.7%	1.04
Rwanda DHS 2010	82.0%	64.2%	57.7%	0.90
Sao Tome Principe DHS 2008	60.8%	51.0%	45.9%	0.90
Senegal DHS 2010	66.2%	38.1%	28.9%	0.76
Senegal MIS 2008	60.4%	34.9%	22.9%	0.66
Sierra Leone 2008 DHS	36.6%	18.8%	19.2%	1.02
Swaziland DHS 2006	4.4%	2.3%	0.3%	0.11
Tanzania DHS 2010	63.8%	46.6%	45.1%	0.97
Tanzania THMIS 2007–2008	39.2%	25.4%	20.3%	0.80
Tanzania THMIS 2011	90.9%	74.5%	68.4%	0.92
Uganda DHS 2011	59.8%	44.7%	35.0%	0.78
Uganda MIS 2009	46.7%	31.6%	25.6%	0.81
Zambia DHS 2007	53.3%	33.9%	23.0%	0.68
Zimbabwe DHS 2005–2006	9.1%	4.8%	2.4%	0.50
Zimbabwe DHS 2010	28.8%	20.2%	8.7%	0.43
**Mean**	**47.1%**	**31.2%**	**27.0%**	**0.81**
**Median**	**50.0%**	**31.6%**	**25.6%**	**0.82**

The range of values for household ownership of ITN was from 3.5% (Guinea 2005) to 90.9% (Tanzania 2011). Median proportion of the *de facto* population with access to an ITN within the household was 31.6%, ranging from 1.5% (Guinea 2005) to 74.5% (Tanzania 2011). Use of an ITN the previous night ranged from 0.3% (Swaziland 2006) to 68.4% (Tanzania 2011). The ratio of use to access ranged from 0.11 (Swaziland 2006) to 1.19 (Madagascar 2011).

**Figure 1:**
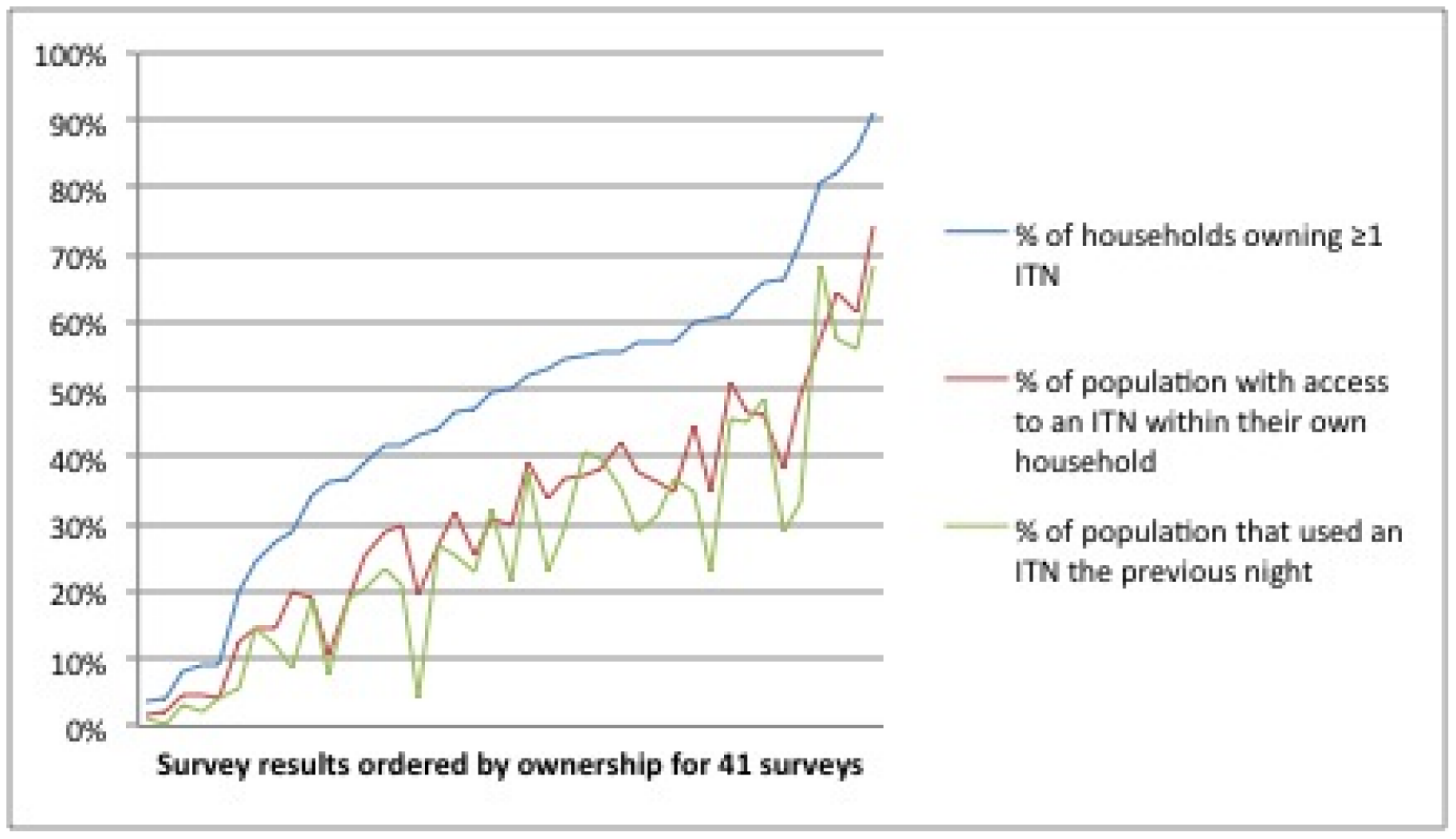
Ownership, access and use of ITNs for all datasets. Survey results are ordered by ownership. Previously, the visual gap between ownership (blue line) and use (green line) made it seem as though the use gap was vast. When use is compared to access (red line), however, a much closer relationship – and narrower gap – is immediately apparent.

**Figure 2:**
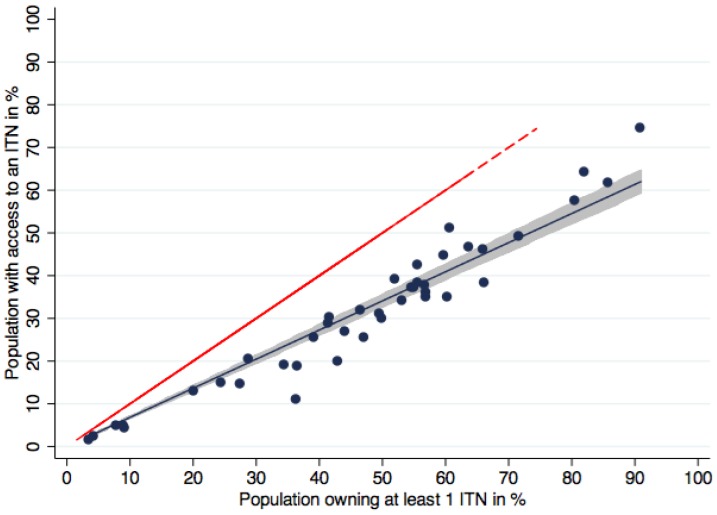
Population with access to an ITN within the household compared to ownership of at least one ITN. Blue dots represent the data points for data sets, the blue line the regression function (fitted values). Shaded area is the 95% confidence interval of the fitted values of population with access to an ITN within the household. Red dashed line represents the equity line where ownership is equal to access. On average, population access was 32% lower than household ownership.

Ownership of ITNs was consistently higher than population access and population use, while access and use tracked more closely as illustrated in Figure 1. Regression analysis showed that there was a close, linear relationship between access and ownership (Figure 2, p<0.0001, R-squared 0.98) with a regression coefficient of 0.68.

### The ratio of use to access

**Figure 3:**
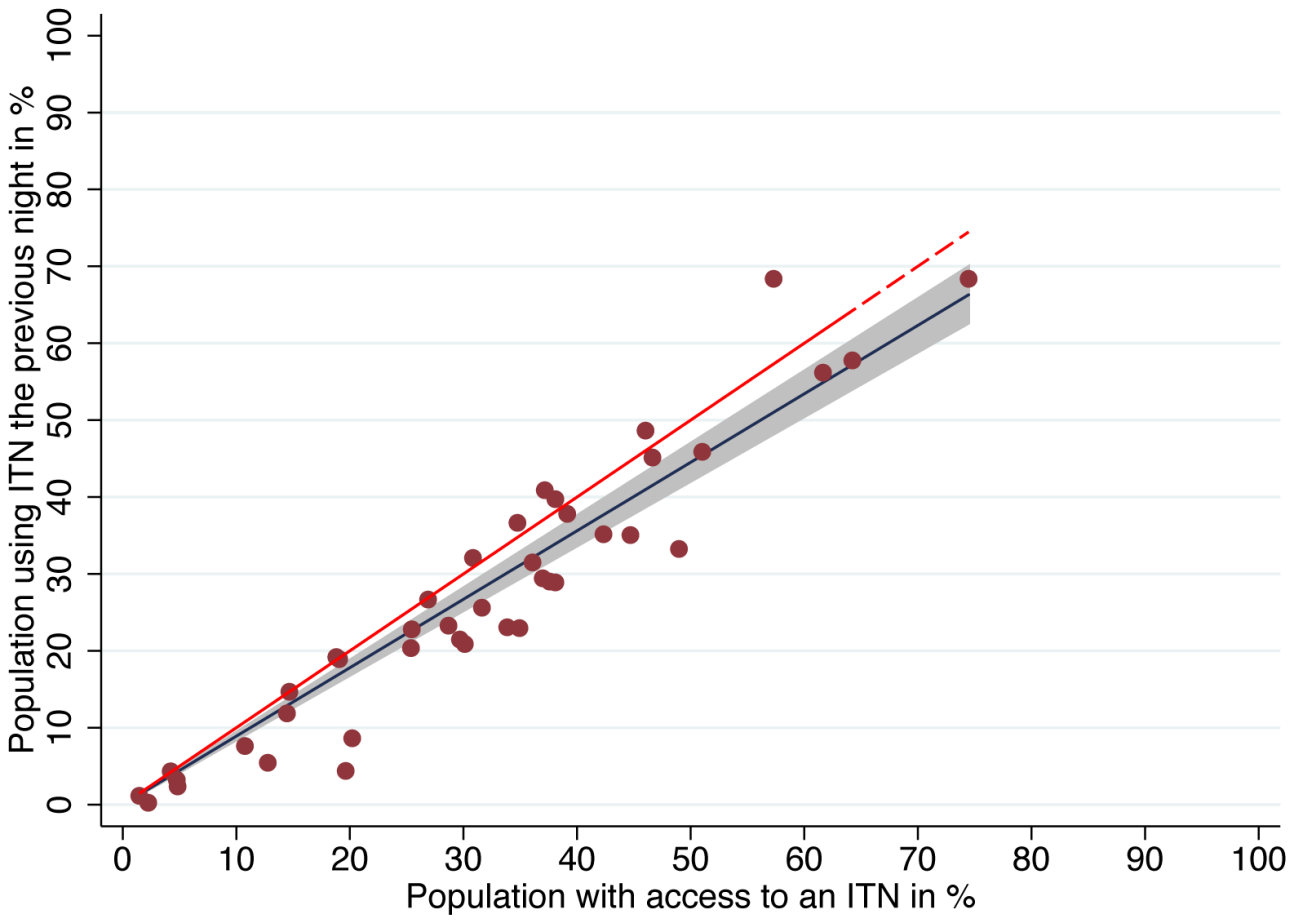
Relationship of ITN use to ITN access. The figure illustrates the linear relationship of use to access. The red dots are proportion of the population that used an ITN the previous night from the survey datasets. The blue line represents the regression line (fitted values). The shaded area is the 95% confidence interval of the fitted values. The red dashed line represents the equity line, where use equals access. On average, 89% of those with access used a net the previous night.

**Figure 4:**
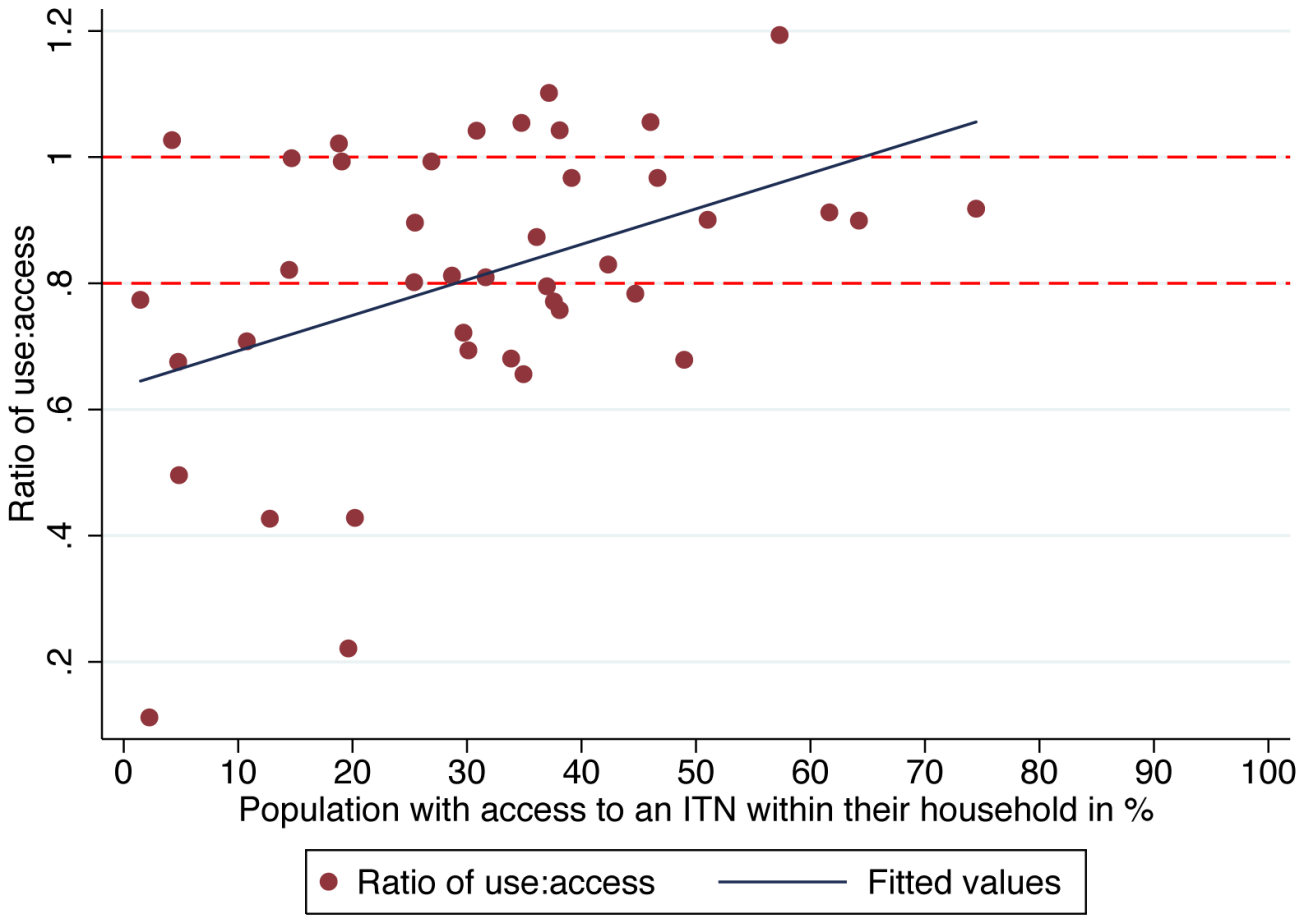
Ratio of use to access by access to an ITN within the household. The ratio of use to access is plotted in red dots, by access. The top red dashed line represents the threshold of 100% use among those with access; the lower dashed line represents a nominal minimum target of 80% use among those with access. The blue line is the fitted values indicating the positive linear relationship between the use to access ratio and access. As access (x-axis) increases, use also increases.

Overall the median proportion of ITN users compared to those with access within the household was high, at 82.1% (Interquartile Range 70.7% to 99.2%) with ten surveys (24%) showing proportions below 70% (range 11.2% to 69.4%) and another eight surveys with a result above 100% (range 102% to 119%) indicating that mean users per net exceeded 2.0 in these cases. Even at population access levels below 50%, a median 80.6% used an ITN given they had access, and this rate increased to 91.2% for access rates >50%. Linear regression of ITN use against access showed an estimated use of 89.0% (95% CI 84.0–93.9) given access (Figure 3) and comparison with a polynomial model confirmed that a linear function was the best fit to the data. However, at lower access values the variation in use was high, then significantly decreased as access rates improved (test for heteroskedasticity p = 0.008), indicating more consistent use of ITNs at higher access rates (Figure 4). For the four surveys where household ownership met Abuja targets (greater than 80%), the mean ratio of ITN use to access was 0.98.

The relationship between the ratio of ITN use to access appeared initially to increase over time, but multivariate regression analysis indicated that the relationship was confounded by increasing access over time, due to the scale up through mass ITN distributions of the past few years.

## Discussion

The newly recommended indicator of population access to an ITN within the household provides a much more appropriate comparison for ITN use than does the household ownership indicator. Previously, when comparing household ownership to population use, it was not possible to determine whether the gap between the two indicators was due to behavioral factors or due to not having enough nets for all the members of the household. Since the two indicators had different denominators, conclusions were difficult to draw. Comparing population ITN use against population ITN access provides a clearer picture of the size of the behavioral gap.

Below 50% access, the median use to access ratio was 80.5%, and above 50% access, the median use to access ratio increased to 91.2%, indicating that at high rates of population access, very few people are not using them. Even at lower levels of population access, use to access ratios above 80% indicate that there is – in general – perhaps only a small amount of room for improvement in net use behavior.

As the population ITN access indicator is calculated by randomly assigning household members to nets, it is not possible to analyze the determinants of non-use for individuals who had access. It is not known whether the individual truly had access or not, due to the randomization process in the population access calculation. This prevents detailed analysis of the determinants of non-use among those with access which might inform BCC planning to improve targeting of messaging to these ‘hold outs’. However, analysis of use rates by age in households with enough nets compared to households without enough nets indicates that those most likely not to be using a net when nets are scarce are adolescents and the elderly [41], as adults and young children tend to be prioritized for net use [9], [14], [17], [20], [42]–[46]. Reported reasons for not using nets when one is available are well documented [21], [24], [47]–[49], and non-use is primarily due to lack of perceived mosquito density and hot nighttime temperatures [21]. Aside from these main subjective reasons, preferences for various design aspects (size, shape, color, texture, density of fabric) have been shown to limit use of nets in some households in Ghana, Ethiopia, and in the Peruvian Amazon [22]–[24], although preferences have not been widely shown to significantly affect ITN use in sub-Saharan Africa. While comparative acceptability and preference studies are useful for determining stated preferences in a given area, they do not indicate whether households would use a non-preferred net just as often, in the absence of their preferred net. Other objective barriers also prevent net use such as its usual user being absent, particularly for funerals [50], being too old or torn, or the net not yet being dry from washing or otherwise unavailable [21], [51], [52].

These results should be considered encouraging for both donors and malaria control program officials, as they show that the vast majority of those who have access to ITNs are using them, and that donor investments are not being wasted. Whether these high use rates are due to the extensive BCC efforts of the past decade, to an increasing familiarity with ITNs [51], or solely to improvements in access is not known. It is likely, despite a dearth of published literature specifically on malaria, that BCC has contributed significantly to the high rates of use, given evidence from Cameroon [27] and Zambia (Boulay, personal communication) that ITN use is significantly associated with exposure to messages about malaria. What is apparent from these data, however, is that as population access increases, the ITN use to access ratio increases, which may indicate a growing social norm of ITN use as ITNs are increasingly available. Ratios of use to access above 100% indicate that more than two people are sharing a net, on average, which should not be surprising considering that multiple children may be sharing both a sleeping space and its ITN, particularly in conditions of ITN scarcity, or in homes where hanging multiple nets is made difficult due to the size or other characteristics of the dwelling or the sleeping rooms' alternative uses [18], [20]. The very low use to access ratios from Namibia, Swaziland, and Niger date from 2006, prior to any scale-up of ITNs; Swaziland benefited from robust IRS operations at the time, while in Niger use dropped dramatically during dry season, when fieldwork was conducted [53].

These findings are in line with other national-level studies [26] that demonstrate that access is the main driver of ITN use. A recent analysis from Nigeria [41] showing that use to access ratios vary considerably between northern and southern Nigeria (0.89 and 0.64, respectively) is already being utilized to focus BCC efforts more strategically in the southern part of the country (Nigeria Malaria Elimination Programme, personal communication). It will be important to look more closely at subnational trends in order to effectively identify and respond to variations in net use within countries. The population access indicator, while it does not allow for individual-level analyses, does allow for analyses at the household level, such as socio-economic status, geographic location, and others. While the use:access ratio provides a better quantification of the behavioral “use gap”, this calculation still does not offer any insights into the reasons why individuals do not use the nets. Future studies will need to include questions that allow these reasons and determinants to be elucidated. The recent Malaria BCC Indicator Reference Guide was designed by RBM partners to help with this and other areas of malaria BCC evaluation [54].

Malaria program officials should continue to work towards closing the access gap by ensuring ways of providing enough nets to all households. Continuous distribution of ITNs through antenatal clinics, immunization programs, school distributions and community distributions, as well as through social marketing and retail sales, provide several options to ensure households can obtain nets between or instead of mass campaigns. At the same time, better understanding of the ITN use gap and the effects of BCC will be necessary to maintain the gains in use and to strengthen the culture of net use that is growing around the continent [51], [55].

## Conclusion

The “net use gap” often referred to by program planners when looking at the standard indicators of household ownership of ITNs and then at population ITN use does not take into account whether there are enough ITNs in the household. On the whole, over 80% of those with access to an ITN within their household reported using an ITN the previous night. This has significant implications for planning behavior change interventions to increase use. These results clearly show that previous interpretations of the net use gap as a failure of behavioral change communication interventions were not justified and that the gap was instead primarily driven by lack of intra-household access. They also demonstrate the usefulness of the newly recommended distinction between use and actual access to ITN.
