# Correction: Recalculating the Net Use Gap: A Multi-Country Comparison of ITN Use versus ITN Access

**DOI:** 10.1371/journal.pone.0161417

**Published:** 2016-08-11

**Authors:** Hannah Koenker, Albert Kilian

The values for household ownership of any ITN for Gabon and Cameroon were incorrect in data set which resulted in several errors in the published article.

In Table 1, the values in the first column for Gabon, Cameroon, and the mean for all countries are incorrect. Please see the corrected Table 1 here.

**Table 1:** Access, use, and ownership of ITNs by survey.

Country | Survey | Year	% of households owning at least 1 ITN	% of population with access to an ITN within their own household	% of population that used an ITN the previous night	Ratio of use to access
Angola MIS 2006–2007	27.5%	14.5%	11.9%	0.82
Angola MIS 2011	34.5%	19.0%	18.9%	0.99
Benin DHS 2006	24.5%	14.7%	14.7%	1.00
Burkina Faso DHS 2010	56.9%	36.1%	31.5%	0.87
Burundi DHS 2010	52.0%	39.1%	37.8%	0.97
Burundi MIS 2012	66.0%	46.0%	48.6%	1.06
Cameroon DHS 2011	18.3%	10.8%	7.6%	0.71
Cote d'Ivoire DHS 2012	71.7%	49.0%	33.2%	0.68
DRC DHS 2007	9.2%	4.2%	4.3%	1.03
Gabon DHS 2012	36.1%	26.9%	26.7%	0.99
Ghana DHS 2008	41.7%	30.1%	20.9%	0.69
Guinea DHS 2005	3.5%	1.5%	1.1%	0.77
Kenya DHS 2008	55.7%	42.3%	35.1%	0.83
Liberia MIS 2009	47.2%	25.4%	22.8%	0.90
Liberia MIS 2011	49.7%	30.8%	32.1%	1.04
Madagascar DHS 2008	57.0%	34.7%	36.6%	1.05
Madagascar MIS 2011	80.5%	57.3%	68.4%	1.19
Malawi DHS 2010	56.8%	37.6%	29.0%	0.77
Malawi MIS 2012	55.0%	37.2%	40.9%	1.10
Mali Anemia & Parasitemia 2010	85.9%	61.6%	56.2%	0.91
Mali DHS 2006	50.0%	29.7%	21.4%	0.72
Mozambique DHS 2011	54.7%	37.0%	29.4%	0.80
Namibia DHS 2006	20.2%	12.8%	5.5%	0.43
Niger DHS 2006	43.0%	19.6%	4.4%	0.22
Nigeria DHS 2008	8.0%	4.8%	3.2%	0.68
Nigeria MIS 2010	41.5%	28.7%	23.3%	0.81
Rwanda DHS 2007–2008	55.6%	38.1%	39.7%	1.04
Rwanda DHS 2010	82.0%	64.2%	57.7%	0.90
Sao Tome Principe DHS 2008	60.8%	51.0%	45.9%	0.90
Senegal DHS 2010	66.2%	38.1%	28.9%	0.76
Senegal MIS 2008	60.4%	34.9%	22.9%	0.66
Sierra Leone 2008 DHS	36.6%	18.8%	19.2%	1.02
Swaziland DHS 2006	4.4%	2.3%	0.3%	0.11
Tanzania DHS 2010	63.8%	46.6%	45.1%	0.97
Tanzania THMIS 2007–2008	39.2%	25.4%	20.3%	0.80
Tanzania THMIS 2011	90.9%	74.5%	68.4%	0.92
Uganda DHS 2011	59.8%	44.7%	35.0%	0.78
Uganda MIS 2009	46.7%	31.6%	25.6%	0.81
Zambia DHS 2007	53.3%	33.9%	23.0%	0.68
Zimbabwe DHS 2005–2006	9.1%	4.8%	2.4%	0.50
Zimbabwe DHS 2010	28.8%	20.2%	8.7%	0.43
**Mean**	**46.5%**	**31.2%**	**27.0%**	**0.81**
**Median**	**50.0%**	**31.6%**	**25.6%**	**0.82**

There are also errors in the graphs for Fig 1, Fig 2, and the caption for Fig 2. Please see the complete, correct graphs and captions for Fig 1 and Fig 2 here.

**Fig 1:**
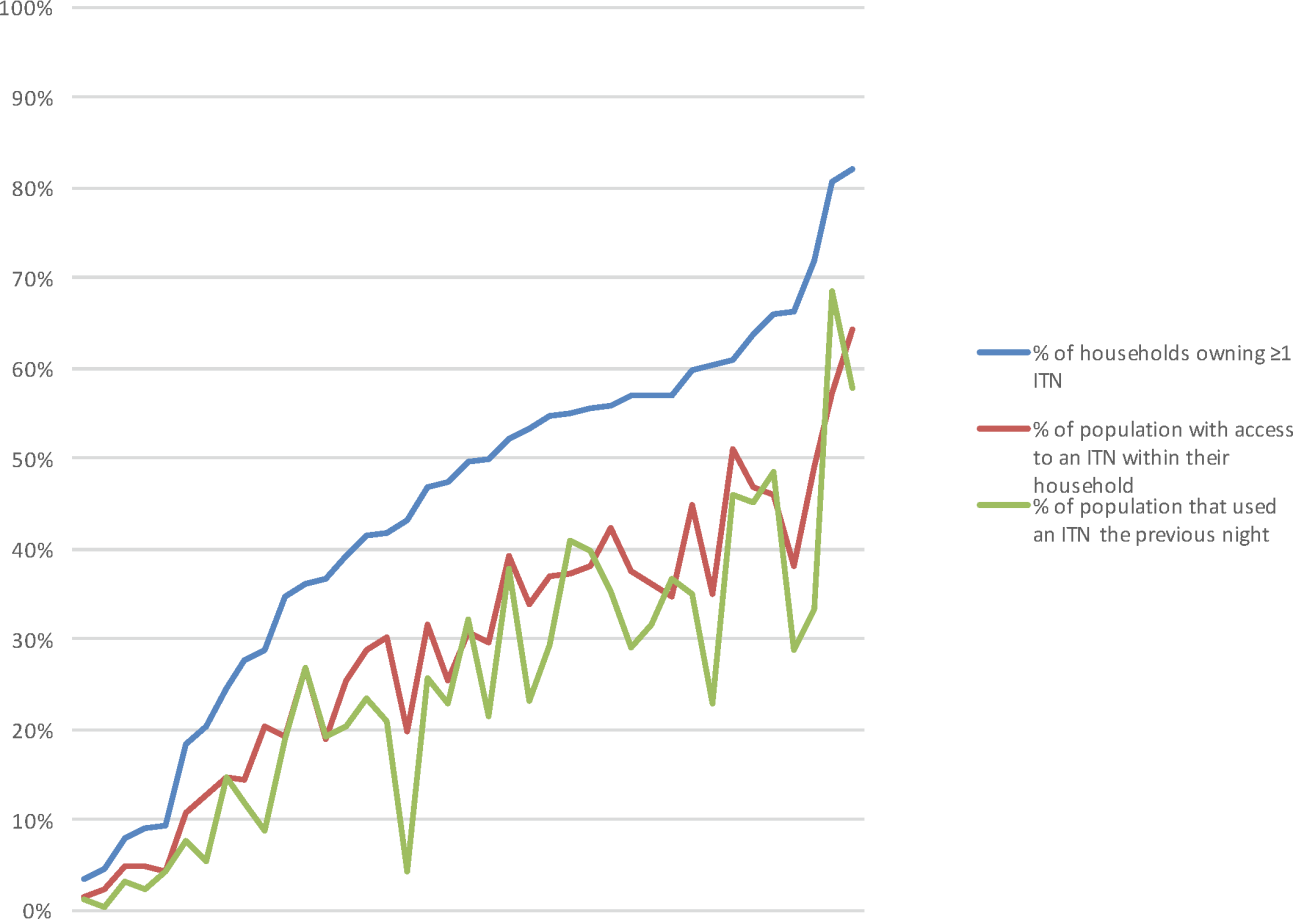
Ownership, access and use of ITNs for all datasets. Survey results are ordered by ownership. Previously, the visual gap between ownership (blue line) and use (green line) made it seem as though the use gap was vast. When use is compared to access (red line), however, a much closer relationship–and narrower gap–is immediately apparent.

**Fig 2:**
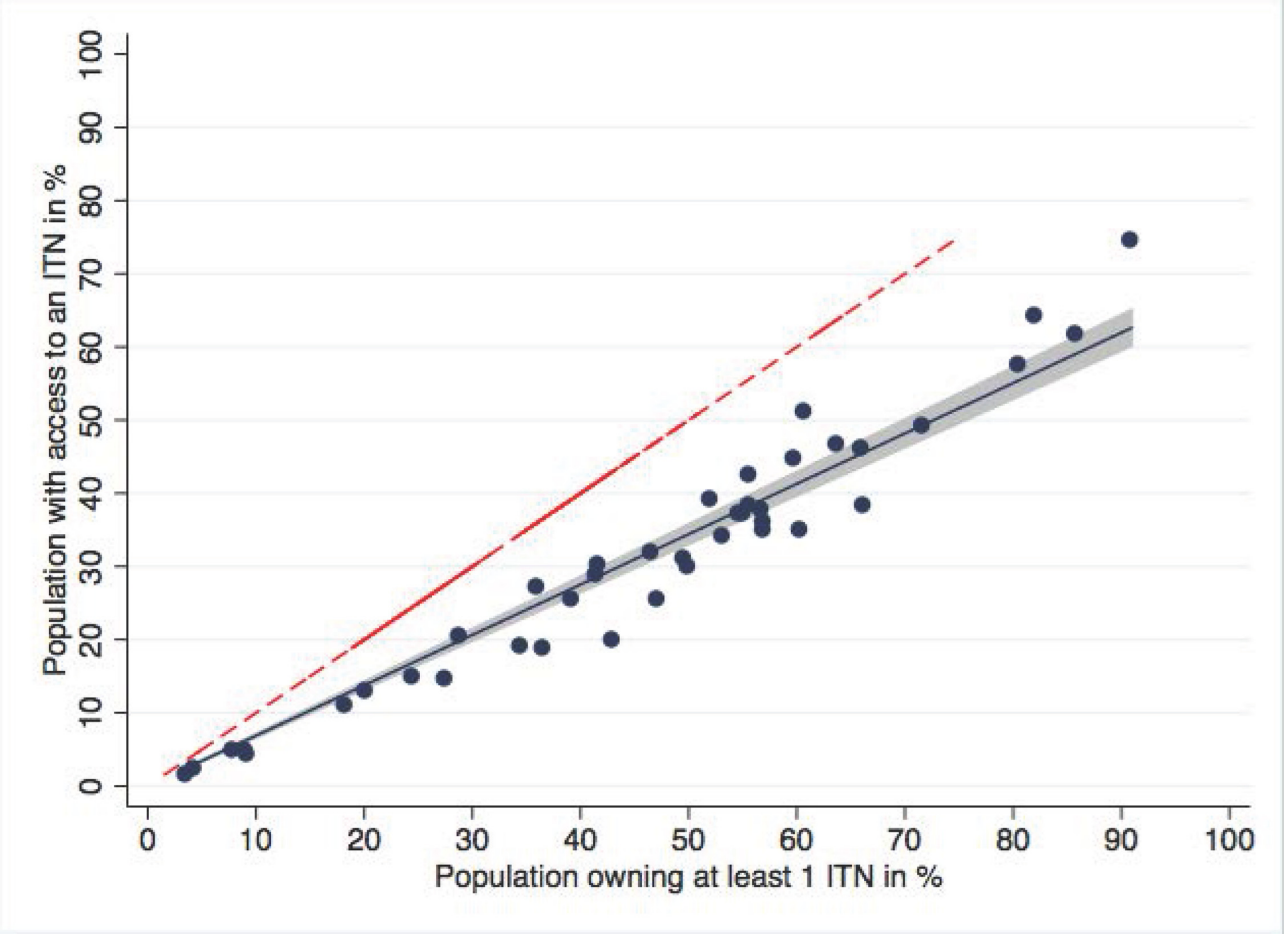
Population with access to an ITN within the household compared to ownership of at least one ITN. Blue dots represent the data points for data sets, the blue line the regression function (fitted values). Shaded area is the 95% confidence interval of the fitted values of population with access to an ITN within the household. Red dashed line represents the equity line where ownership is equal to access. On average, population access was 31% lower than household ownership.

There is an error in the ninth sentence of the Results. The correct sentence is: Regression analysis showed that there was a close, linear relationship between access and ownership (Fig 2, p<0.0001, R-squared 0.98) with a regression coefficient of 0.69.

There is also an error in the eleventh sentence of the Results. The correct sentence is: Even at population access levels below 50%, a median 80.6% used an ITN given they had access, and this rate increased to 91.2% for access rates >50%. Linear regression of ITN use against access showed an estimated use of 89.0% (95% CI 84.1–94.0) given access (Figure 3) and comparison with a polynomial model confirmed that a linear function was the best fit to the data.
